# Sixteen years of change in the global terrestrial human footprint and implications for biodiversity conservation

**DOI:** 10.1038/ncomms12558

**Published:** 2016-08-23

**Authors:** Oscar Venter, Eric W. Sanderson, Ainhoa Magrach, James R. Allan, Jutta Beher, Kendall R. Jones, Hugh P. Possingham, William F. Laurance, Peter Wood, Balázs M. Fekete, Marc A. Levy, James E. M. Watson

**Affiliations:** 1 Ecosystem Science and Management Program, University of Northern British Columbia, Prince George, British Columbia, Canada V2N 4Z9; 2 Centre for Conservation and Biodiversity Science, The University of Queensland, St Lucia Queensland 4072, Australia; 3 Centre for Tropical Environmental and Sustainability Science, College of Science and Engineering, James Cook University, Cairns, Queensland 4878, Australia; 4 Wildlife Conservation Society, Global Conservation Program, Bronx New York 10460, USA; 5 Ecosystem Management, ETH Zurich, 8092 Zurich, Switzerland; 6 Doñana Biological Station (EBD-CSIC), Avd. Américo Vespucio s/n, Isla de la Cartuja, 41092 Sevilla, Spain; 7 School of Geography, Planning and Environmental Management, University of Queensland, St Lucia Queensland 4072, Australia; 8 Department of Life Sciences, Imperial College London, Silwood Park, Ascot, Berkshire SL5 7PY, UK; 9 Department of Civil Engineering, The City College of New York, CUNY Environmental CrossRoads Initiative, City University of New York, New York, New York 10007, USA; 10 Center for International Earth Science Information Network, Columbia University, Palisades, New York 10964, USA

## Abstract

Human pressures on the environment are changing spatially and temporally, with profound implications for the planet's biodiversity and human economies. Here we use recently available data on infrastructure, land cover and human access into natural areas to construct a globally standardized measure of the cumulative human footprint on the terrestrial environment at 1 km^2^ resolution from 1993 to 2009. We note that while the human population has increased by 23% and the world economy has grown 153%, the human footprint has increased by just 9%. Still, 75% the planet's land surface is experiencing measurable human pressures. Moreover, pressures are perversely intense, widespread and rapidly intensifying in places with high biodiversity. Encouragingly, we discover decreases in environmental pressures in the wealthiest countries and those with strong control of corruption. Clearly the human footprint on Earth is changing, yet there are still opportunities for conservation gains.

## Abstract (web summary)

Habitat loss and urbanization are primary components of human impact on the environment. Here, Venter *et al.* use global data on infrastructure, agriculture, and urbanization to show that the human footprint is growing slower than the human population, but footprints are increasing in biodiverse regions.

## Graphical abstract

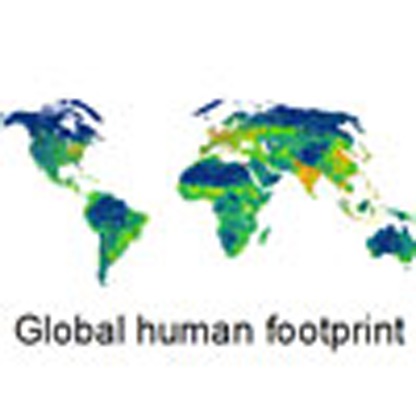

Humanity and nature form a coupled system^1^. The ecological capital and ecosystem services provided by nature underpin our social and economic systems^2^, and we in turn apply pressure on these natural systems through our extraction of natural resources, the proliferation of our infrastructures and our conversion of natural habitats to production land uses^3^. There is mounting evidence that human demands on natural systems are accelerating and could be undermining the stability of these systems^4^. A pervasive failure to mitigate these impacts is now resulting in widespread biodiversity declines^5^,^6^ and reductions in the benefits humans receive from natural systems^2^.

Understanding the spatial and temporal patterns in human pressures on the environment provides a foundation for mitigating environmental damage in sensitive or ecologically valuable areas. Human pressures on the environment, commonly referred to as threats to biodiversity, are the actions taken by humans with the potential to harm nature^7^. Recent advances in remote sensing have allowed unprecedented advances in mapping human pressures from habitat conservation through time, especially for forested landscapes^8^. However, many forms of human pressure on the environment, such as our extensive roads and pasture lands, are harder to detect than outright habitat conversation, and often overlooked by space-borne satellites^9^. Cumulative threat mapping aims to surmount this limitation by including a range of human pressures within a framework that couples top-down remote sensing with data collected bottom-up via surveys^10^.

A range of cumulative threat maps have been developed at regional^11^ and global scales^12^,^13^. The Human Footprint, first released in 2002 based on data from the early 1990s (ref. 14), is unique for considering eight human pressures globally, making it the most complete and highest-resolution globally consistent terrestrial data set on cumulative human pressures on the environment.

Terrestrial maps of cumulative human pressures have proved to be better predictors of the range sizes of species than their biological traits, such as body size and trophic level^15^, and to be a strong predictor of modern range collapses^16^, the threat status of species^17^, site-scale species richness^18^, and species population size and dispersal ability^19^. Cumulative pressures are also associated with invasibility^20^. With calls for rapid action to prevent collapse of planetary ecological systems^21^, we need to better understand spatial and temporal trends in human pressures and their related consequences, so we can act accordingly.

Here we use the human footprint framework to compile remotely sensed and bottom-up survey information on eight variables measuring the direct and indirect human pressures on the environment in 1993 and 2009. We included data on the following: (1) extent of built environments; (2) crop land; (3) pasture land; (4) human population density; (5) night-time lights; (6) railways; (7) roads; and (8) navigable waterways. These pressures were weighted according to estimates of their relative levels of human pressure following Sanderson *et al.*^14^, and then summed together to create the standardized human footprint for all non-Antarctic land areas. The primary aims of this study are to update the original human footprint map to provide a contemporary view of human pressures, and to create the first temporally consistent maps of the human footprint, such that patterns of change over time can be analysed. In addition to these aims, we perform a number of preliminary analyses determinants of important spatial and temporal patterns in the human footprint, and we discuss a number of remaining unanswered questions for subsequent focused analyses. Broadly, our analyses reveal that the human footprint is widespread and rapidly increasing, especially in tropical ecoregions and other locations rich in biodiversity. Wealthy nations and those with strong control of corruption showed some signs of improvements, yet this is overshadowed by the fact that 71% of global ecoregions saw marked (>20%) increases in their human footprints.

## Results

### The global human footprint

**Figure 1:**
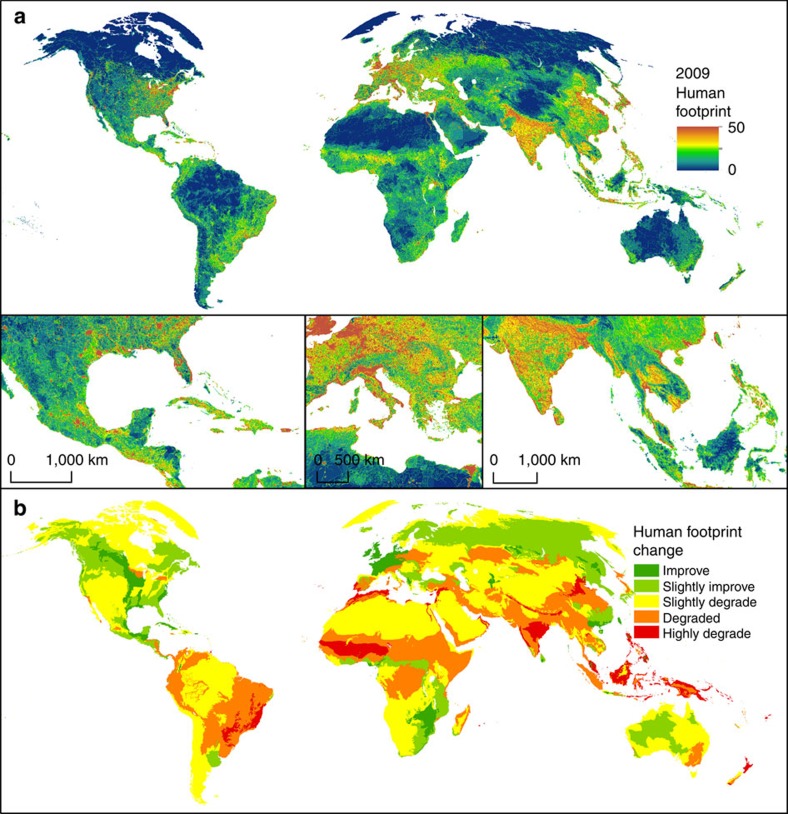
Maps showing the current state and recent change in the global human footprint. (**a**) The global human footprint map for 2009 using a 0–50 cool to hot colour scale, and (**b**) absolute change in average human footprint from 1993 to 2009 at the ecoregion scale^74^. Data on human footprint change are summarized by ecoregions to allow for interpretation of broad patterns. Inset panels in **b** show focal regions that span the full breadth of the human footprint pressure scale.

We find that in 2009, the world's land areas had an area-weighted average human footprint score of 6.16 (out of a maximum of 50; Table 1), which is an increase of 9% from 1993 levels. These pressures show strong spatial variation. The highest pressure biomes include the temperate broadleaf forests of Western Europe, eastern United States and China, and the tropical dry forests of India and parts of Brazil, and parts of Southeast Asia's tropical moist forest (Fig. 1a). Areas of no measureable human footprint (as mapped by our eight pressures) persisted over ∼27% of the world's non-Antarctic land area in 1993. But these areas of intact habitats have decreased rapidly over the past two decades, with 23 million km^2^ (9.3%) experiencing an incursion of human pressures. The remaining pressure-free lands are concentrated in the boreal and tundra biomes, the Sahara, Gobi and Australian deserts, and the most remote moist tropical forests of the Amazon and Congo Basins.

Averaging over the 823 ecoregional boundaries provides an overview of change in the human footprint from 1993 to 2009 (Fig. 1b). Most ecoregions are undergoing increases in average footprint values (*n*=573), especially in tropical regions such as Southeast Asia and eastern Brazil, while some ecoregions appear to be improving (*n*=223), primarily in temperate zones such as North America and Western Europe. These patterns would indicate that change may be underway in divergent trajectories across space, with important ramifications for biodiversity.

**Figure 2:**
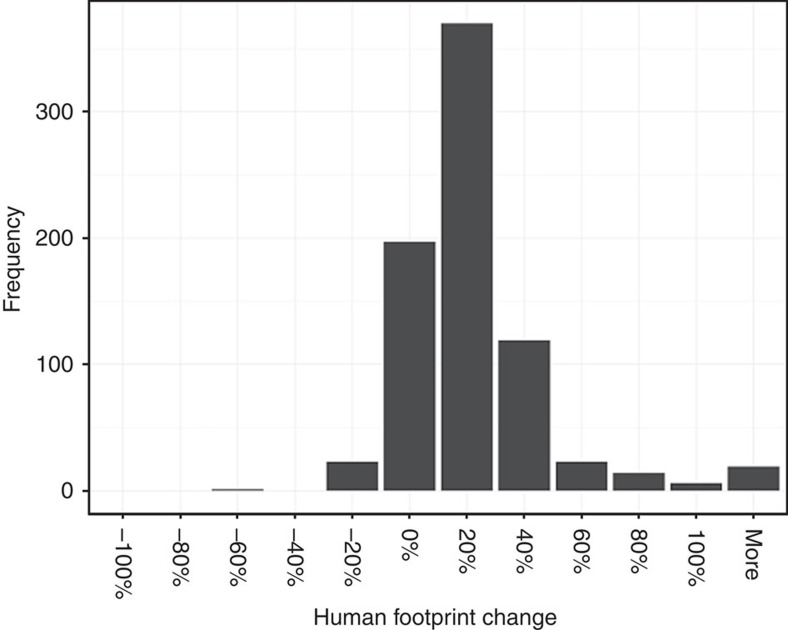
Human footprint change summarized by terrestrial ecoregions. Histogram shows per cent change in average human footprint scores for the world's 823 non-Antarctic ecoregions.

Only 3% of 772 ecoregions saw declines in human pressure >20%, whereas 71% saw average increases of 20% or more (Fig. 2). Examining ecoregions of least 1,000 km^2^ in extent, average human footprint decreased in some temperate forests and grasslands in the Northern hemisphere (for example, Northern transitional alpine forests, −65%; Canadian aspen forests and parklands, −38%; Central tall grasslands, −30%; and Caledon conifer forests, −30%) and in selected tropical montane forests (for example, peninsular Malaysian montane rain forest, −40%; Belizean pine forests, −33%; and Sri Lankan montane rain forests, −32%.) In contrast, pressures more than doubled in 20 ecoregions globally, for example, in numerous North American tundra ecosystems (for example, Torngat Mountain tundra, >+10,000%; Davis Highlands, +5,090%; and Baffin coastal tundra, +1,591%), across most forested ecoregions in New Guinea (for example, New Guinea mangroves, +151%; Southern New Guinea lowland rain forests, +321%; and New Britain–New Ireland lowland rain forests, +141%) and in selected Neotropical rain forests (for example, Purus várzea,+161%; and Japura-Solimoes-Negro moist forests, +101%). Admittedly, the largest percentage increases were seen in ecoregions with a low base level of human pressure, but these increases reinforce the point about decline of wilderness over the study period.

Our validation of the human footprint map shows there is strong agreement between the human footprint measure of pressure and pressures scored by visual interpretation of high-resolution images (Supplementary Fig. 1 and Supplementary Note 1). The root mean squared error^22^ for 3,114 validation 1 km^2^ plots was 0.15 on a normalized 0–1 scale. The Kappa statistic^23^ was 0.737, also indicating good agreement between the human footprint and the validation data set. Of the 3,114 1 km^2^ validation plots, the human footprint scored 94 of them 20% higher than the visual score (false positive) and 263 of them 20% lower (false negative). The remaining 2,757 plots (88.5%) were within 20% agreement. While this represents good agreement, it appears from the higher false-negative rate that the human footprint is to some extent susceptible to false negatives, where pressures are actually present in locations where the human footprint maps them as absent. The maps should therefore be considered as conservative estimates of human pressures on the environment.

### Human footprint and agricultural suitability

**Figure 3:**
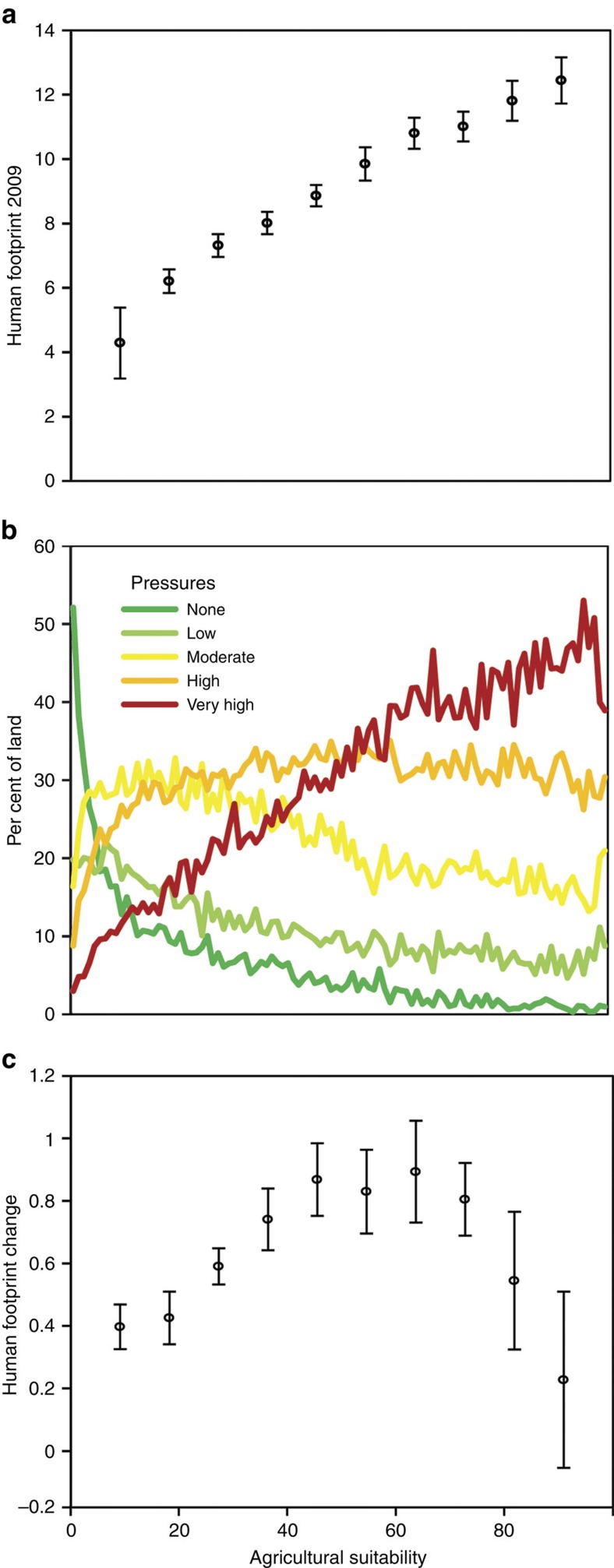
Relationships between the human footprint and suitability of land for agriculture. The suitability of land and its relationship to (**a**) the mean and s.d. of the human footprint in 2009, (**b**) the spatial extent of five human footprint bins and (**c**) the mean change and s.d. in the human footprint. The thresholds that define the five human footprint bins in **b** are set such that each bin covers a similar proportion of the world's land areas in 2009, with higher pressure bins represented by warmer colours.

We used data on the suitability of land for agriculture^24^ to evaluate the relationship between the human footprint trends and agricultural suitability. Agricultural suitability ranges from 0% suitable to 100% suitable based on climatic and soil constraints^24^. While the patterns are complex, the suitability of lands for agriculture appears to be a major determinant of the intensity, extent and temporal trends of pressures across the globe. A correlation between agriculture suitability and cumulative human pressures is expected, yet the strength of this relationship is surprising. We find a near continuous linear increase in cumulative pressures with increasing agricultural suitability (Fig. 3a), with 93.8% of the variation in average footprint values explained by agricultural suitability alone (linear model, *t*-value= 38.77, *P*<0.001).

To measure the spatial extent of the human footprint in 2009, we bin land areas into five classes of human pressure such that each bin encompasses a similar proportion (∼20%) of the planet, and label these bins, ‘no pressure, mean human footprint=0'; ‘low pressure, human footprint=1–2; ‘moderate pressure, human footprint=3–5'; ‘high pressure, human footprint=6–11'; and ‘very high pressure, human footprint=12–50'. We find that lands of no pressures dominate in places that are fully unsuitable for agriculture (for example, deserts), but are then rapidly replaced by moderate- and high-pressure bins in areas even marginally suitable for agriculture (Fig. 3b). Pressure-free lands are almost totally lacking in locations which are at least 60% suitable for agriculture, and the extent of very-high-pressure bins increases linearly until it becomes the dominant category at around the 60% suitable for agriculture mark. The near-total dominance of very-high-pressure lands in locations most suitable for agriculture has important implications for the ecological communities associated with these areas, which we explore in the next section.

While human pressures show a consistent positive association with agricultural suitability in 2009, the change in human pressures since 1993 follows an inverted-U pattern (Fig. 3c). Similar to the least suitable areas, the most suitable areas showed little change since 1993, potentially as these areas were already saturated with pressures by then. On the other hand, moderately suitable areas, ranging from 40 to 80% suitability have experienced a rapid increase in human footprint since 1993. This pattern is likely explained by the expansion of agriculture and other human pressures into these more marginal lands.

### Human footprint and biodiversity

The interaction of abiotic conditions and evolutionary history has made some places on Earth teem with life, while others are more biologically poor. Places that house large concentrations of overlapping species are particularly important for biodiversity conservation^25^, as their loss contributes disproportionately to the current extinction crisis. With increasingly complete data sets on the distributions of plants and animals^26^, we now have a reasonable idea of where species are concentrated^26^,^27^. However, comparatively little is known about the pressures facing these concentrations of biodiversity.

To determine the pressures within areas high in biodiversity we use three complementary measures of areas with high concentrations of species. First, we use biodiversity hotspots^28^, which are defined as areas having at least 1,500 endemic vascular plants and at least 70% of their natural vegetation cleared, and they are therefore expected to be under high pressure. To complement these plant-based areas, we also map high concentrations of vertebrates by overlaying the 21,059 range maps of terrestrial birds^29^, mammals^30^ and amphibians^30^ onto a 30 km grid of the world's land areas. We map high concentrations of these vertebrates as places with at least 569 vertebrates, which is the threshold that defines the richest 10% of the planet. Threatened species are most likely to go extinct in the near term, and we map threatened vertebrate concentrations as the areas with at least 14 threatened vertebrates, which is the threshold necessary to encompass 10% of land areas.

**Figure 4:**
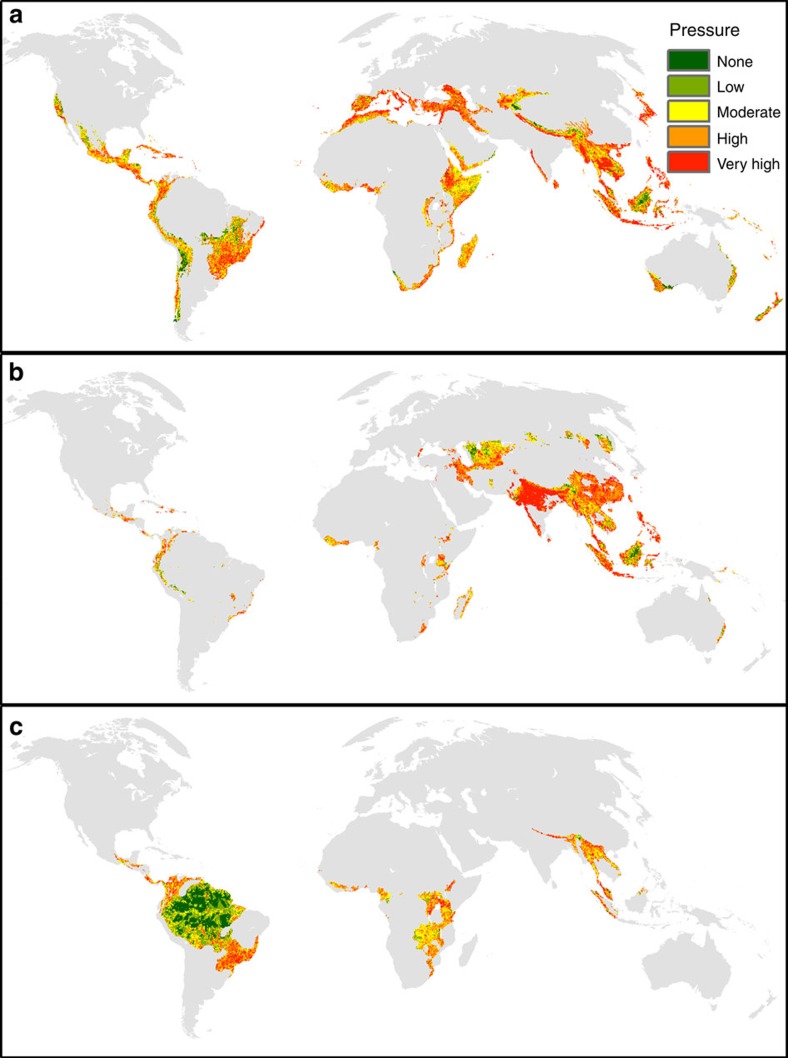
The extent of the human footprint within important areas for biodiversity. The distribution of human footprint intensity bins across (**a**) biodiversity hotspots^28^, (**b**) high concentrations of threatened vertebrates and (**c**) high concentrations of all vertebrates. High concentrations are the 10% of land areas encompassing the highest global concentrations of either threatened vertebrates or all vertebrates^29^,^30^. Green represents areas with a human footprint score of 0, and warmer colours represent higher-pressure bins.

We find that biodiversity hotspots and areas that contain high numbers of threatened vertebrate species are under high human pressure (Fig. 4a,b). High- and very-high-pressure bins cover the majority of these biodiversity valuable lands. For biodiversity hotspots, only small areas of no or low human footprint still exist in the Western Australia, Tropical Andes, Northern Cerrado and Central Asian Mountain Biodiversity hotspots. For areas that contain high numbers of threatened vertebrates species, some low-pressure land still exists in the heart of Borneo and in the Central Asia Desserts between the Caspian and Aral Seas. However, areas of no pressure cover <3% of the world's biodiversity hotpots and 2% of threatened vertebrate concentrations.

A more encouraging picture emerges when looking at the places on the planet that have the highest overall richness of birds, mammals and amphibians (Fig. 4c). The Amazon basin falls almost entirely within the 10% most species-rich land on Earth. While the Amazon is bisected in parts by navigable waterways and roads, and is increasingly encroached by settlements and industrial agriculture (especially in the southern region), much of the Amazon is still free of human pressures (Fig. 4c). Globally, there is an even distribution of human footprint categories across the areas that contain high numbers of vertebrates, which indicates remaining opportunities for a proactive approach to conservation that maintains these most-specious of ecosystems in their natural condition before pressures infiltrate them^31^.

**Figure 5:**
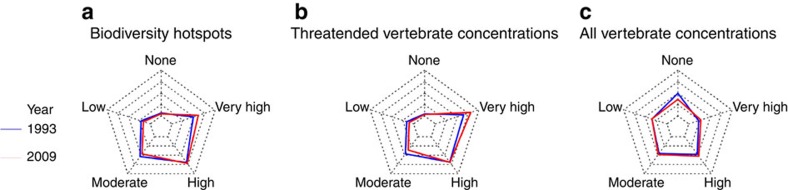
Change in human footprint within important areas for biodiversity. The extent of human footprint intensity bins across (**a**) biodiversity hotspots^28^, (**b**) high concentrations of threatened vertebrates and (**c**) high concentrations of all vertebrates for 1993 (blue lines) and 2009 (red lines).

While vertebrate concentrations still have extensive areas of no human pressure, these areas are disappearing at an alarming rate (Fig. 5). Our results show a rapid decline in no-pressure lands in vertebrate richness concentrations since 1993 (Fig. 5c). Human pressures expanded into intact areas holding the highest concentrations of vertebrates at a rate faster than any transition in any other area. Moreover, there were large increases in areas of high- and very-high-pressure bins, which are likely to have the greatest impacts on native biodiversity. Biodiversity hotspots and threatened vertebrate hotspots also experienced similar increases in these heavily impacted areas (Fig. 5a,b).

### The human footprint and socio-economic change

The pressures underlying the human footprint are all linked in some way to socio-economic activities. These pressures include our urban centres, which are the powerhouses of the global economy, agricultural lands, transport networks and the people who are ultimately responsible for all economic output. With a human footprint that is already near ubiquitous, especially in places suitable for agricultural and high in biodiversity, and forecasts for significant population and economic growth^32^, it would be encouraging if the human footprint is not deterministically linked to socio-economic growth.

To investigate this, we compared the growth in the human footprint from 1993 to 2009 with population and economic changes over the same period. We note that while the human population has increased by 23% (ref. 33) and the world economy has grown by 153% (ref. 33), the human footprint has increased by just 9%. It appears as though the global human economy is increasing its efficiency in the use of land resources when measured in terms of human footprint per person or per dollar gross domestic product (GDP), which is in line with findings from other studies^34^.

**Figure 6:**
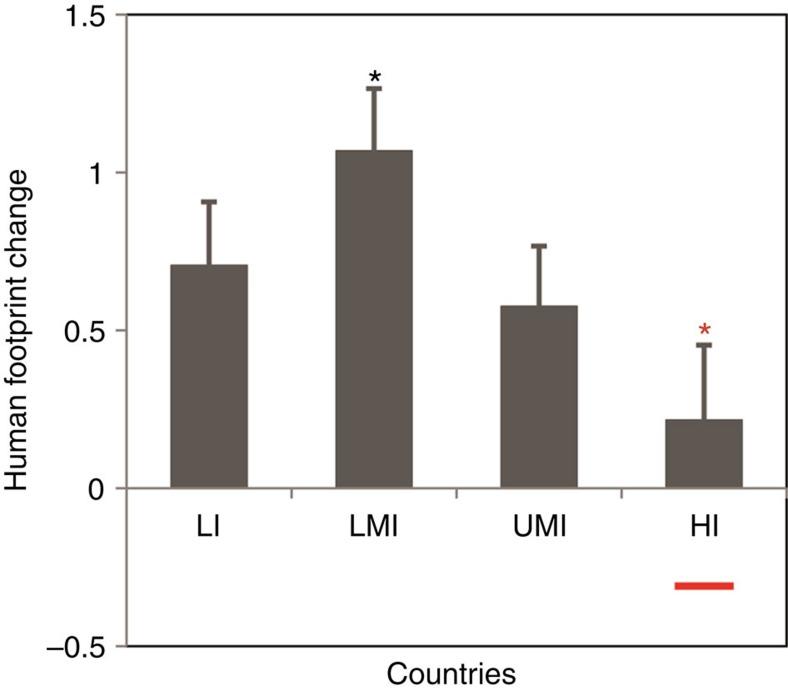
Change in human footprint across countries grouped by income level. Average change in the human footprint over the 1993 to 2009 period for low-income (LI, *n*=36), lower-middle-income (LMI, *n*=42), upper-middle-income (UMI, *n*=44) and high-income (HI, *n*=51) countries^33^. The horizontal red bar below the HI column shows the average change for the wealthiest countries (*n*=24), defined as those with >$30,000 per person GDP at PPP in 2009. Black bars show standard errors and coloured asterisks denote statistical differences.

To further investigate this relationship, we quantified the change in the human footprint for countries grouped by income level. We find an inverted-U-shaped relationship for human pressure across income categories (Fig. 6), with lower-middle-income countries undergoing the greatest increase in human footprint and high-income countries undergoing the least. Moreover, we find that footprint trajectories have actually reversed in the wealthiest nations (Fig. 6, red bar). This trend is not entirely unexpected considering that environmental Kuznets curve theory posits an inverted-U-shaped relationship between economic growth and environmental degradation^34^.

The decrease in human pressures in wealthy countries may indicate a more environmentally sustainable future, with increased wealth and urbanization leading to reduced human footprint for some countries^34^. However, it is important to determine if this trend is driven by measures of socio-economic and governance conditions or rather by patterns of trade that allow some countries to transfer their demand for food and raw materials to other countries. For example, almost 40% of beef produced in the Amazon is not consumed domestically but instead exported for consumption in European Union countries^35^.

We investigate the link between trade and the human footprint by focusing on the top 50 percentile of countries for GDP growth per person (*n*=73 with data available). For these rapidly expanding economies, we ask whether their highly divergent environmental outcomes, in terms of their human footprint, can be explained by variation in their rates of international trade, measured as the net trade in agricultural and forestry products, or instead by proxies for socio-economic transformation and governance. Crop and pasture lands are the greatest driver of land conversion^36^, and timber plantations cover 140 million hectares, with at least a further 340,000 hectares of selective logging annually^37^. Surveying trade in these sectors should therefore provide a reasonable approximation of whether a country is exporting its environmental pressures through international trade. This method does have a number of limitations, which are discussed further in the following section.

Of the 73 rapidly expanding economies, 47 experienced increased human pressure and 26 experienced decreased human pressure (Table 2). Net trade does not appear to be driving this divergence, as economically growing countries with declining human pressure tended to be net exporters (US$1.3 billion or 17 million tons) while economically growing countries with increasing human pressure tended to be net importers (US$−1.8 billion or −7 million tons; Table 2). Instead, environmentally improving countries are characterized by higher rates of urbanization, human development (a composite measure of health and education) and control of corruption (Table 2). A parsimonious general linear model explaining the environmental trajectories of the expanding economies includes their rate of urbanization and control of corruption (Supplementary Table 1), with only corruption being statistically significant (Supplementary Fig. 2).

## Discussion

The human footprint presents a spatially explicit, temporally consistent and quantitative measure of the *in situ* direct and indirect human pressures on the environment. Pressures are chosen to represent some of the most important actions taken by humans with the potential to harm local natural systems. These pressures include measures of land cover change, such as urban and agricultural land uses, infrastructures, such as railways and electric infrastructure, and access into natural areas, such as via roads and navigable waterways. Our study presents an update to the original human footprint map^14^, and extends previous work by providing the first set of temporally intercomparable human footprint maps. Moreover, our validation of the human footprint map found strong agreement with an independently derived measure of human pressures.

We find strong relationships between the severity, extent and expansion of the human footprint and the suitability of land for agriculture. The high pressures facing much of the planet highlights the urgent need for enhanced conservation interventions. Some of the hardest decisions about protecting natural landscapes must be taken within the planet's most biologically valuable regions. While remote sensing has estimated biodiversity hotspots to have around 15% natural vegetation remaining^38^, our results suggest that opportunities for conservation may be much constrained, with only 3% of these areas currently free of human pressures. This result indicates that maintaining biodiversity will require extensive restoration to remove and mitigate existing pressures^39^. Restoration may be particularly beneficial where pressures have arisen only recently, as the time-lags before biodiversity declines could mean that many species could still be saved^40^. Our analyses also highlight the importance of the Amazon basin as a highly specious and still largely intact system, but one now susceptible to accelerated pressures following recent policy changes^41^,^42^.

We found that some countries, particularly wealthy countries and those associated with high urbanization and low corruption, have been undergoing rapid economic growth while simultaneously reducing their human footprint. Most encouragingly, these countries are net exporters of agricultural and forestry products, and by this measure are not simply exporting their demand for food and fibre (and the associated local pressures) to other countries. These results are encouraging for the future as countries continue to expand economically and increase their rates of urbanization. We caution that these findings must be viewed as preliminary for two primary reasons. First, we are using the value and volume of trade as a proxy for the international ‘trade' in human pressures. This combined measure of trade may hide differences among agricultural and forestry products in their production footprint. Second, we did not account for the broader suite of exportable environmental pressures, such as those associated with mineral products, manufactured goods and energy^43^.

Moreover, our work is subject to three general limitations. First, like all attempts to map cumulative pressures we did not fully account for all human pressures. Some of the missing and static pressures, such as invasive species and pollution, may be closely associated with pressures we did consider^44^, and therefore their inclusion may not affect our overall results. Second, a lack of available data resulted in three of our pressures being static through time, which would cause an underestimation of human footprint expansion if these pressures expanded at a higher than average rate. We were able to determine the sensitivity of our results to treating pasture lands, which are one of the most spatial extensive human pressures. Using dynamic pasture data at the national scale changed our national level results on average by 2%, and did not change our findings qualitatively (Supplementary Table 2). Third, the human footprint measures the pressure humans place on nature, not the realized state or impacts on natural systems or their biodiversity^7^. Significant scope exists to determine how natural systems respond to cumulating human pressures, and if nonlinearity or thresholds exist where pressures lead to accelerated impacts. In light of these limitations, we make all of our maps available for download^73^,^74^, including all our individual pressures should data users wish to include new data or alter the use of existing data to create alternate maps of human pressure.

The human footprint continues to expand on Earth, but at an overall rate that is slower than the underlying rates of population and economic growth. These results are profound for nature and humanity. Still, the near ubiquity of the pressures we exert on nature highlight the enormous challenges involved in achieving continued socio-economic growth without widespread environmental degradation.

## Methods

### Overview

To create the human footprint we adopted the methods developed by Sanderson *et al.*^14^. To facilitate comparison across pressures we placed each human pressure within a 0–10 scale (not all pressure range across the full 0–10 scale, details on the weightings for each pressure are provided in the flowing sections) and acquired data for the early 1990s (on average 1993) and 2009. The human pressures we considered included the following: (1) the extent of built environments; (2) crop land; (3) pasture land; (4) human population density; (5) night-time lights; (6) railways; (7) roads; and (8) navigable waterways. These pressures were weighted according to estimates of their relative levels of human pressure following Sanderson *et al.*^14^ and summed together to create the standardized human footprint for all non-Antarctic land areas. Pressures are not intended to be mutually exclusive, and many will co-occur in the same location. Three pressures only had data from a single time period, and these are treated as static and excluded from all trend analyses (Table 1). We tested the sensitivity of our results to these static data, and to the scoring scheme, results below. We used ArcGIS 10.1 to integrate spatial data on human pressures. Analyses were conducted in Goode's homolosine equal area projection at the 1 km^2^ resolution, yielding ∼134.1 million pixels for Earth's terrestrial surface. For any grid cell, the human footprint can range between 0 and 50.

### Built environments

Built environments are human-produced areas that provide the setting for human activity. In the context of the human footprint, we take these areas to be primarily urban settings, including buildings, paved land and urban parks. Built environments do not provide viable habitats for many species of conservation concern, nor do they provide high levels of ecosystem services^45^,^46^. As such, built environments were assigned a pressure score of 10.

To map built environments, we use the Defense Meteorological Satellite Program Operational Line Scanner (DMSP-OLS) composite images, which gives the annual average brightness of 30 arcsec (∼1 km at the equator) pixels in units of digital numbers^47^. These data are provided for each year from 1992 to 2012. We extracted data for the years 1994 (1993 was excluded due to anomalies in the data) and 2009, and all years were then inter-calibrated to facilitate comparison across the years^48^. Using the DMSP-OLS data sets, we considered pixels to be built if they exhibited a calibrated digital number (DN) >20. We selected this threshold based on a global analysis of the implications of a range of thresholds for mapped extent of cities^49^, and visual validation against Landsat imagery for 10 cities spread globally.

### Population density

Many of the pressures humans impose on the environment are proximate to their location, these include pressures such as disturbance, hunting and the persecution of non-desired species^50^. Moreover, even low-density human populations with limited technology and infrastructure developments can have significant impacts on biodiversity, as evidenced by the widespread loss of various taxa, particularly mega fauna, following human colonization of previously unpopulated areas^51^.

Human population density was mapped using the Gridded Population of the World data set developed by the Centre for International Earth Science Information Network^52^. The data set provides a ∼4 km × ∼4 km gridded summary of population census data for the years 1990 and 2010, which we downscaled to match the 1 km^2^ resolution of the other data sets. For all locations with more than 1,000 people·per km, we assigned a pressure score of 10. For more sparsely populated areas, we logarithmically scaled the pressure score using


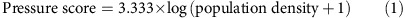


### Nighttime lights

The high sensitivity of the DMSP-OLS^47^ data set provides a means for mapping the sparser electric infrastructure typical of more rural and suburban areas. In 2009, 79% of the lights registered in the DMSP-OLS data set had a DN <20, and are therefore not included in our built environments layers. However, these lower DN values are often important human infrastructures, such as rural housing or working landscapes, with associated pressures on natural environments.

To include these pressures, we used the inter-calibrated DMSP-OLS layers^47^. The equations for inter-calibrating across years are second-order quadratics trained using data from Sicily, which was chosen as it had negligible infrastructure change over this period, where DN average roughly 14. For our purposes, DN values of 6 or less were excluded from consideration before inter-calibration of data, as the shape of the quadratic function leads to severe distortion of very low DN values. The inter-calibrated DN data from 1994 were then rescaled using an equal quintile approach into a 0–10 scale. The thresholds used to bin the 1994 data were then used to convert the 2009 data into a comparable 0–10 scale.

### Crop and pasture lands

Crop lands vary in their structure from intensely managed monocultures receiving high inputs of pesticides and fertilizers to mosaic agricultures such as slash and burn methods that can support intermediate levels of many natural values^53^,^54^. For the purposes of the human footprint, we focused only on intensive agriculture because of its greater pressure on the environment, as well as to circumvent the shortcomings of using remotely sensed data to map mosaic agriculture globally, namely the tendency to confound agriculture mosaics with natural woodland and savannah ecosystems^55^.

Spatial data on remotely sensed agriculture extent in 1992 were extracted from the UMD Land Cover Classification^56^, and for 2009 from GlobCover^57^. Although intensive agriculture often results in whole-scale ecosystem conversion, we gave it a lower score than built environments because of less impervious cover.

Pasture lands cover 22% of the Earth's land base or almost twice that of agricultural crops^58^, making them one of the most extensive direct human pressure on the environment. Land grazed by domesticated herbivores is often degraded through a combination of fencing, intensive browsing, soil compaction, invasive grasses and other species, and altered fire regimes^59^. We mapped grazing lands for the year 2000 using a spatial data set that combines agricultural census data with satellite derived land cover to map pasture extent^58^. We assigned pasture a pressure score of 4, which was then scaled from 0 to 4 using the per cent pasture for each 1 km^2^ pixel.

### Roads and railways

As one of humanity's most prolific linear infrastructures, roads are an important direct driver of habitat conversion^60^. Beyond simply reducing the extent of suitable habitat, roads can act as population sinks for many species through traffic-induced mortality^61^. Roads also fragment otherwise contiguous blocks of habitat, and create edge effects such as reduced humidity^62^ and increased fire frequency that reach well beyond the roads' immediate footprint^63^. Finally, roads provide conduits for humans to access nature, bringing hunters and nature users into otherwise wilderness locations^64^.

We acquired data on the distribution of roads from the global roads open access data set (gROADS)^65^, and excluded all trails and private roads, which were inconsistently mapped. The data set is the most comprehensive publicly available database on roads, which has compiled nationally mapped road data spanning the period 1980–2000. We mapped the direct and indirect pressure of roads by assigning an pressure score of 8 for 0.5 km out for either side of roads, and access pressures were awarded a score of 4 at 0.5 km and decaying exponentially out to 15 km either side of the road.

While railways are an important component of our global transport system, their pressure on the environment differs in nature from that of our road networks. By modifying a linear swath of habitat, railways exert direct pressure where they are constructed, similar to roads. However, as passengers seldom disembark from trains in places other than rail stations, railways do not provide a means of accessing the natural environments along their borders. To map railways we used the same data set as was used in the original footprint^66^, as no update of this data set or alternate source has been developed. The direct pressure of railways was assigned a pressure score of 8 for a distance of 0.5 km on either side of the railway.

### Navigable waterways

Like roads, coastlines and navigable rivers act as conduits for people to access nature. While all coastlines are theoretically navigable, for the purposes of the human footprint we only considered coasts^66^ as navigable for 80 km either direction of signs of a human settlement within 4 km of the coast. We chose 80 km as an approximation of the distance a vessel can travel and return during daylight hours if travelling at 40 km h^−1^. As new settlements can arise to make new sections of coast navigable, coastal layers were generated for the years 1994 and 2009.

Large lakes can act essentially as inland seas, with their coasts frequently plied by trade and fishing vessels. On the basis of their size and visually identified shipping traffic and shore side settlements, we treated the great lakes of North America, Lake Nicaragua, Lake Titicaca in South America, Lakes Onega and Peipus in Russia, Lakes Balkash and Issyk Kul in Kazakhstan, and Lakes Victoria, Tanganyika and Malawi in Africa as we did navigable marine coasts.

Rivers were considered as navigable if their depth was >2 m and there were signs of human settlements within 4 km of their banks, or if contiguous with a navigable coast or large inland lake, and then for a distance of 80 km or until stream depth is likely to prevent boat traffic. To map rivers and their depth we used the hydrosheds (hydrological data and maps based on shuttle elevation derivatives at multiple scales)^67^ data set on stream discharge, and the following formulae from^68^,^69^:


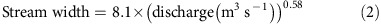


and


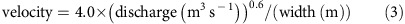


and,


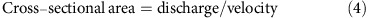


and


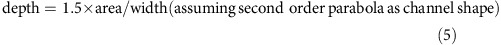


Navigable river layers were created for the years 1994 and 2009, and combined with the navigable coasts and inland seas layers to create the final navigable waterway layers. The access pressure from navigable water bodies was awarded a score of 4 adjacent to the water body, decaying exponentially out to 15 km.

### Validating the human footprint map

High-resolution images (median=0.5 m) were used to visually interpret human pressures at 3,560 1 km^2^ sample points randomly located across the Earth's non-Antarctic land areas (Supplementary Fig. 1). For the visual interpretation, the extent of built environments, crop land, pasture land, roads, human settlements, infrastructures and navigable waterways was recorded using a standard key for identifying these features (Supplementary Note 1). Shape, size, texture and colour were important characteristics for identifying human pressures on the environment. Interpretations were also marked as certain or not certain, and the year and the resolution of the interpreted image were recorded. The 344 uncertain points were discarded, leaving 3,116 validation points. The human footprint score for each point was determined in ArcGIS, and the visual and human footprint scores were then normalized to a 0–1 scale. The human footprint score was considered as a match to the visual score if they were within 20% of one another on the 0–1 scale.

### Sensitivity to static data sets and scoring

Three data sets (pasture lands, roads and railways) were treated as static pressures in our human footprint maps, as temporally inter-comparable data were not available for these pressures at a resolution ammenable to inclusion in the human footprint. If these pressures changed at rates that were higher or lower relative to the dynamic data sets, it could mean that our estimates of change in the human footprint were similarly lower or higher than actual change. We were able to test the sensitivity of maps to static data sets for pasture lands. We acquired data on national level changes in pasture extent from 1993 to 2009 from the United Nations Food and Agricultural Organization^1^.

Given that these data are national scale, we were able to determine how the analyses of change across countries would be perturbed if our static pasture data were replaced with dynamic data from the United Nations Food and Agricultural Association (UN FAO). Using the FAO data we were able to estimate the likely changes in the average contribution of pasture land pressures to changes in the human footprint across countries. This was done by multiplying the 1993 human footprint pasture pressure data by the country-level change in pasture extent from the FAO. We found inclusion of dynamic pasture pressures in this way did not change our national-scale analyses of changes in the human footprint. Our estimates of national level change in human footprint were very similar using the static or dynamic pasture data (Pearson's *R*^2^=99.8%, *P*<0.0001) with an average perturbation from the static data with the dynamic data of just ±2.6%, and Upper-middle-income countries still underwent the greatest increases and high-income countries underwent the least (Supplementary Table 1). We could not perform similar analyses for railways or roads, as changes in these linear infrastructures over time are simply not available, even at the national scale.

As described in the preceding sections, the eight pressures were scaled onto a 0–10 scale according to estimates of their relative levels of human pressure following Sanderson *et al.*^14^, before summing together to create the standardized human footprint maps. We adopted the same scaling methods as Sanderson *et al.*^14^, as the original human footprint map has proven to be a strong predictor of a wide range of ecological phenomena, lending support to the scoring scheme. Similar to the sensitivity analyses for static data sets, we tested the sensitivity of our national-scale results to this scoring scheme.

We achieved by first determining the contribution of each of the eight pressures to the overall human footprint score for each country. We then randomly perturbed the score or ‘weighting' for each pressure up by 50%, down by 50% or keep it the same. After this random perturbation, we calculated the new national-scale average human footprint score for each country by multiplying the old score by the random perturbation from, and then summed across pressures. The proportional change in national-scale human footprint was calculated by comparing the original and new human footprint values. Finally, we calculated the relative proportional change in national-scale human footprint by dividing the proportion change observed for a country by the global-scale change induced by the scoring perturbation. These steps were repeated 100 times.

We found that a 50% perturbation to the scoring of each pressure led to on average a 14.5% change in each country's national-scale human footprint. These national-scale changes also led to overall global-scale changes in human footprint values. When removing this global effect and focusing on only the relative changes across countries (such as would be done for the results contained in Fig. 6), we find that the 50% perturbations to the scores led to on average a 7.5% relative change in the national-scale human footprint values. These results demonstrate that national level human footprint values, especially when evaluating how countries compare relative to one another, are robust to how pressures are scored.

### The human footprint national-level change

We compiled a number of national-scale data sets to determine if over-the-horizon consumption, socio-economic transition, urbanization or governance can explain the difference in footprint trajectories among the most rapidly expanding economies. Rapidly developing economies were considered to be the top 50 percentile of countries for GDP at purchasing power parity growth per person over the 1993 to 2009 period (*n*=73). Over-the-horizon consumption was measured as the trade balance (exports minus imports) for all agricultural (including crops and livestock) and forestry products in 2009, extracted from UN FAO^70^. Economic transition was measured in terms of economic development (2009 GDP per capita at PPP^33^) and human development (Non-income Human Development Index, HDI^71^. The non-income HDI takes into account the average achievements of a country for health and education. The degree a country has urbanized was measured in terms of the proportion of its population that lives in urban areas in 2009 (ref. 33). Overall governance capacity was measured in terms of a country's control of corruption^72^, and as a more direct measure of environmental governance, we used the proportion of a country's terrestrial area that has been set aside in protected areas^33^. We excluded all countries smaller than 1,000 km^2^ and those for which data were not available, leaving us with a 146 countries.

To explain the divergent environmental trajectories for the most rapidly expanding economies (countries within top 50 percentile for GDP at PPP per person change between 1993 and 2009) we fitted a general linear model at the country level, including the following variables: country area; GDP at PPP per person in 2009; control of corruption; proportion of country under protection; net trade in agricultural and forestry products (calculated as the sum of the value of agricultural and forestry exports minus that of imports); and the proportion of population in urban areas and non-income HDI. The proportion of urban population and non-income HDI was highly correlated (Spearman's rho=0.72) and they were therefore never included in the same models. We generated all possible subsets of the full model containing all variables and selected the most parsimonious one based on their Akaike information criterion (AIC) score. We also performed the same tests measuring trade in kilograms instead of dollars, but found that it did not alter our results.

### Data availability

The 1 km^2^ resolution human footprint maps are stored in the Dryad Digital Repository (doi:10.5061/dryad.052q5)^73^, and may also be freely accessed through the Socioeconomic Data and Applications Center website (http://www.ciesin.org/). From Dryad the files may be downloaded as a single 7-zip file archive (7-Zip.org), which contains individual GeoTIFF data sets, an excel file with the validation data and a PDF with the validation key. The GeoTIFFs include the human footprint maps for 1993 and 2009, as well 14 additional GeoTIFFs of the processed data for each of the eight pressures from each time step. The individual pressure layers are provided should data users wish to rework these data to create alternate maps of human pressure for their particular needs or region. These data are described in ref. 74

## 

## Additional information

**How to cite this article:** Venter, O. *et al.* Sixteen years of change in the global terrestrial human footprint and implications for biodiversity conservation. *Nat. Commun.* 7:12558 doi: 10.1038/ncomms12558 (2016).

## Supplementary Material

Supplementary Information Supplementary Figures 1-2, Supplementary Tables 1-2 and Supplementary Note 1

## Figures and Tables

**Table 1:** Human pressures used to construct the human footprint (HF).

Data set	Timing	Years	Mean HF score	
			1993	2009
Built environments	Dynamic	1994, 2009	0.17	0.19
Crop lands	Dynamic	1992, 2005	0.79	0.96
Pasture lands*	Static	2000	0.51	0.47
Population density	Dynamic	1990, 2010	2.10	2.32
Night lights	Dynamic	1993, 2009	0.29	0.36
Railways	Static	1960s–1990s	0.15	0.15
Major roadways	Static	1980–2000	1.32	1.32
Navigable waterways	Dynamic	1993, 2009	0.33	0.38
All combined	NA	NA	5.67	6.16

HF, human footprint; NA, not applicable.

Static data are available for only one time period.

^*^Pasture lands' global averages vary across years as pasture is not permitted to overlap with crop or urban lands, which are dynamic data sets.

**Table 2:** Variables used to explain human footprint change across countries.

Variables	HF decreasing		HF increasing	
	Mean	S.d	Mean	S.d
Net trade (million $)	1,278	11,727	−1,765	14,310
Net trade (million tons)	16.78	74.72	−7.36	63.02
GDP ($ per capita)	29,784	15,159	25,145	16,745
Urban (% population)	76.64	12.50	69.60	16.89
HDI (non-income)	0.84	0.07	0.79	0.09
Control of corruption	1.02	1.06	0.26	0.93
Protected (% land)	13.61	8.67	14.62	11.02

HF, human footprint.

Values presented are means and s.d.'s for variables used to explain HF change for 73 rapidly growing economies. Data are summarized for countries where HF is decreasing (environmental improvement; *n*=26) and where HF is increasing (environmental deterioration; *n*=47).
